# “Para-retinal” Vector Administration into the Deep Vitreous Enhances Retinal Transgene Expression

**DOI:** 10.1016/j.omtm.2020.06.015

**Published:** 2020-06-24

**Authors:** Yong Zeng, Ryan Boyd, Joshua Bartoe, Henry E. Wiley, Dario Marangoni, Lisa L. Wei, Paul A. Sieving

**Affiliations:** 1 National Eye Institute, National Institutes of Health, Bethesda, MD, USA; 2 Charles River Laboratories, Matawan, MI, USA; 3 Department of Ophthalmology, University of Pennsylvania, Philadelphia, PA, USA; 4 Center for Ocular Regenerative Therapy; Department of Ophthalmology, University of California at Davis, Sacramento, CA, USA

**Keywords:** AAV vector, AAV8, ocular delivery, intravitreal delivery

## Abstract

Intravitreal administration for human adeno-associated vector (AAV) delivery is easier and less traumatic to ocular tissues than subretinal injection, but it gives limited retinal transduction. AAV vectors are large (about 4,000 kDa) compared with most intraocular drugs, such as ranibizumab (48 kDa), and the large size impedes diffusion to reach the retina from the usual injection site in the anterior/mid-vitreous. Intuitively, a preferred placement for the vector would be deep in the vitreous near the retina, which we term “para-retinal” delivery. We explored the consequences of para-retinal intravitreal delivery in the rabbit eye and in non-human primate (NHP) eye. 1 h after para-retinal administration in the rabbit eye, the vector concentration near the retina remained four times greater than in the anterior vitreous, indicating limited vector diffusion through the gelatinous vitreous matrix. In NHP, para-retinal placement showed greater transduction in the fovea than vector applied in the mid-vitreous. More efficient retinal delivery translates to using lower vector doses, with reduced risk of ocular inflammatory exposure. These results indicate that para-retinal delivery yields more effective vector concentration near the retina, thereby increasing the potential for better retinal transduction in human clinical application.

## Graphical Abstract

**Figure:**
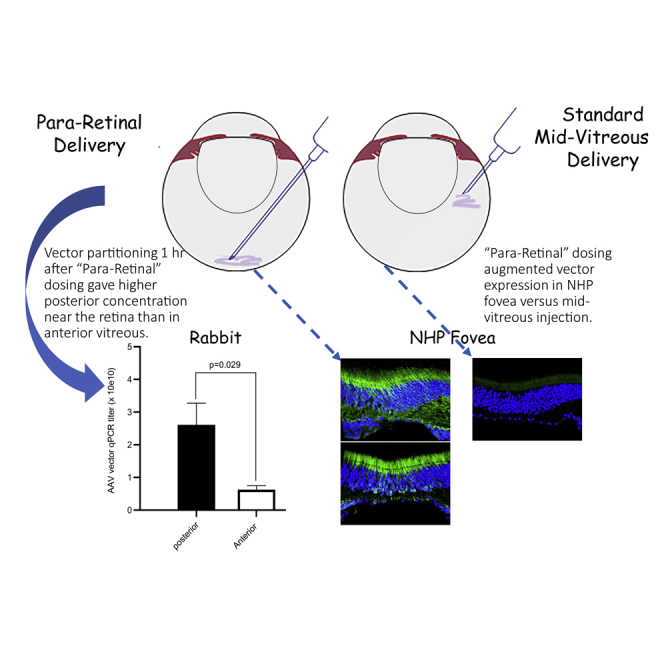

## Teaser

The authors explored an alternate approach to standard intravitreal administration called “para-retinal” delivery where AAV vectors were placed deep into the vitreous near the retina surface. This modification increased vector concentration in the posterior vitreous in rabbit eyes, minimized vector losses, and enhanced foveal transgene expression in NHP eyes.

## Introduction

The eye is advantageous for exploring gene therapy, as the small closed compartment requires minimal vector quantity and is somewhat protected against immune system activation.^1^ Vector is usually delivered to the retina by subretinal or intravitreal injection. Subretinal application, which is used for voretigene neparvovec-rzyl (Luxterna) has the advantage of placing the vector adjacent to target retina cells, including the photoreceptors and retinal pigment epithelium (RPE), which frequently are affected by retinal diseases. Unfortunately, subretinal administration requires surgical manipulation of retinal tissue, and vector distribution is limited to the region near the injection site.^2^ The procedure itself requires local retinal detachment of photoreceptors from the RPE, which may cause immediate or lasting retinal damage.^2^ Intravitreal application is minimally traumatic and can reach a larger expanse of the retina, as used for the Gensight LHON vector, which targets retinal ganglion cells and axons at the retina surface. However, standard intravitreal application yields poor transduction efficiency for reaching the photoreceptors and RPE.^3^

While considerable effort currently is being directed to modifying the adeno-associated vector (AAV) capsid to improve the potency for retinal transduction following standard intravitreal injection,^3^, ^4^, ^5^ we were interested in the physical limitations of AAV diffusion through the vitreous cavity to reach the retinal surface. Exploratory ocular retinal therapies are often developed in the tiny mouse eye but then face the challenge of scaling to the much larger human eye with 800 times more vitreous volume (https://prometheus.med.utah.edu/).^6^ Further, the geometry of the mouse eye is substantially different than human, as the vitreous depth (the space between the posterior lens and the retina) is less than 1 mm in mouse (http://marclab.org/wp-content/uploads/2013/04/Schematic-mouse-rat-human-eye-size.pdf), compared with 16 mm in human.^6^ Hence intravitreal injection in mouse places the vector immediately adjacent to the retina, whereas in human the vector must diffuse through the vitreous to reach the retina after intravitreal injection. The large size of the AAV virus (20–25 nm; 3,700 kDa)^7^^,^^8^ impedes rapid diffusion, and capsid surface charges may further slow the movement to the retina surface from the vitreous injection site.^9^

We used a simple technique called “para-retinal administration” to apply the vector (AAV8-CMV-EGFP) near the retina surface by an intravitreal approach in rabbit eyes and then in NHP eyes (myc-tagged AAV8-retinoschisin [RS1] vector equivalent to the vector for our X-linked retinoschisis human trial^10^). This method significantly improved the distribution of vector near the retina of injected rabbit eyes even at 1 h after administration, and we found that transgene expression in the NHP retina was improved by para-retinal versus standard mid-vitreous injection.

## Results

### Results of Para-retinal Delivery into Rabbit Eyes

**Figure 1:**
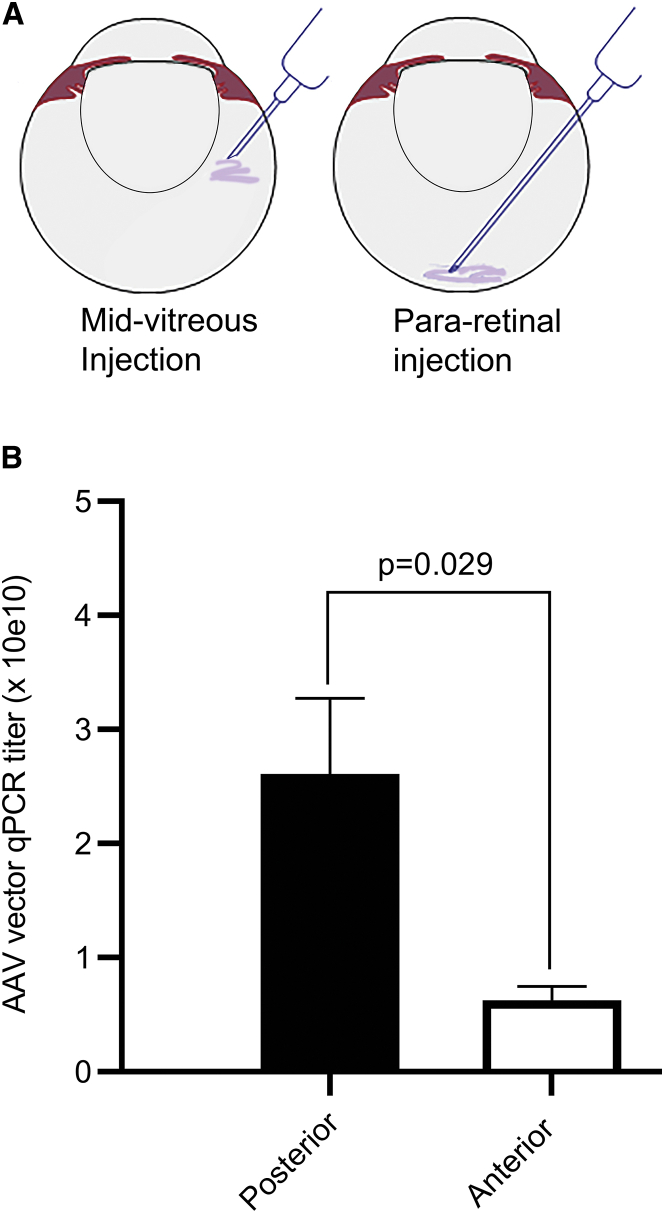
Vector Partitioning in Rabbit Vitreous 1 h after Para-Retinal Injection of AAV8-CMV-*EGFP* (A) The cartoon demonstrates the location of the needle tip for a standard intravitreal injection in the rabbit eye versus para-retinal delivery near the retina. (B) Relative vector concentration 1 h after para-retinal injection (dose of 1.5 × 10^11^ vg per eye). Vitreous was frozen, removed from the globe and tissues, and hemi-sectioned into posterior and anterior segments. AAV titers were determined by qPCR analysis. Titers were significantly higher in the posterior region near the retina (2.61 × 10^10^ vg/mL) versus anterior vitreous (0.63 × 10^10^ vg/mL; p = 0.029, n = 15 eyes). Error bars are SEM.

The rabbit study assessed whether para-retinal delivery of AAV8-CMV-EGFP vector gave higher local concentration that persisted temporarily in the posterior vitreous compared to the anterior vitreous near the lens. For para-retinal administration, the needle tip was advanced close to the retinal surface and vector was released slowly (Figure 1A, right). Dosing was performed on three sets of rabbits, and data were used from all eyes dosed by deep vitreous para-retinal injections (n = 15 eyes) with none excluded.

The vitreous was collected 1 h after para-retinal injection, and vector concentration was determined for the posterior and anterior portions (results in Figure 1B). Vector titers were about four times greater in posterior vitreous near the retina (2.61 × 10^10^ vg/mL, n = 15 eyes) versus the anterior vitreous near the lens (6.31 × 10^9^ vg/mL, n = 15; p = 0.03). This means that even 1 h after para-retinal application, the vector remained relatively concentrated in the deeper vitreous, and the greater vector concentration would augment entry into the retina.

### Results of Para-retinal Delivery into NHP Eyes

**Figure 2:**
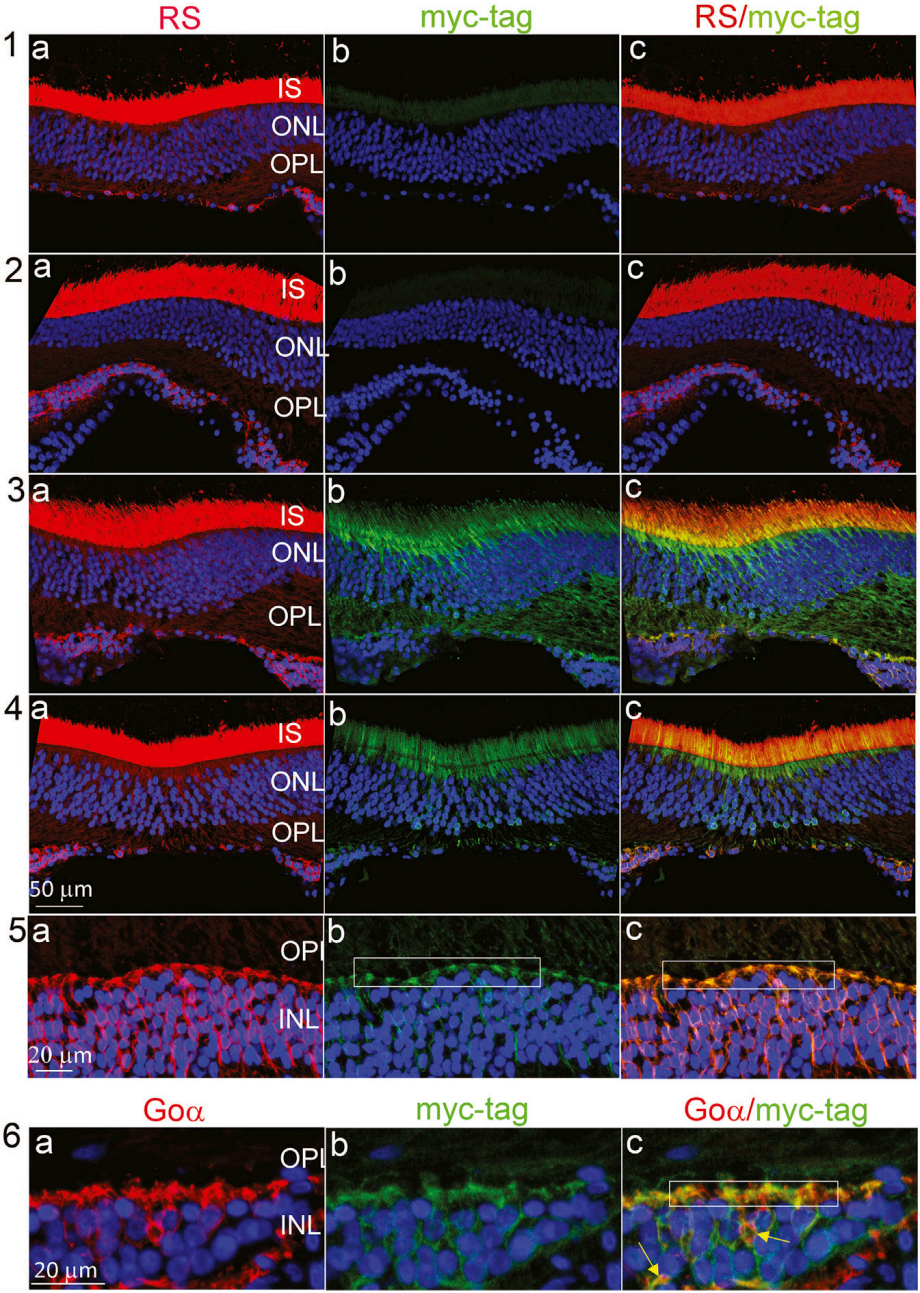
Immunohistochemistry of NHP Fovea NHP fovea immunofluorescence images with counterstaining of anti-myc-tag/retinoschisin (RS1) and anti-myc-tag/G_0_α, following intravitreal injection of AAV8-RS1/myc vector at 3e11 vg/eye (rows 1, 3–5) or excipient (row 2). Left column (1a–5a) shows RS1 labeling, which marks both endogenous RS1 and exogenous hRS1 from the vector. The middle column (1b–5b) myc-tag labeling marks only exogenous hRS1 expression. Row 1: eye received mid-vitreous AAV8-RS1/myc injection. Row 2: eye received excipient. Rows 3 and 4: two eyes each received para-retinal AAV8-RS1/myc injection. Row 5: higher magnification of the images taken from the fovea of the eye in row 3 that received para-retinal vector injection. The myc-tagged RS1 colocalizes with endogenous RS1 in the inner segments (ISs) and the outer plexiform layer (OPL) synapses onto bipolar cells (synapses shown at higher magnification in 5b and 5c, rectangles). Little or no myc-tagged RS1 is detected after mid-vitreous AAV8-RS1 injection (1b). No myc-tagged hRS1 is detected after excipient injection (2b). Row 6: counterstaining with anti-myc-tag and G_0_α (ON bipolar cell and synaptic marker) confirms colocalization to bipolar dendritic tips (6c, rectangular) and cells (6c, yellow arrows). To improve details of colocalization, we balanced the color channels only in (1c)–(6c) (20% increase in green channel and 20% decrease in red channel). ONL, outer nuclear layer; INL, inner nuclear layer. Scale bar (rows 1–4) is 50 μm; scale bar (rows 5 and 6) is 20 μm. Error bars are SEM.

**Figure 3:**
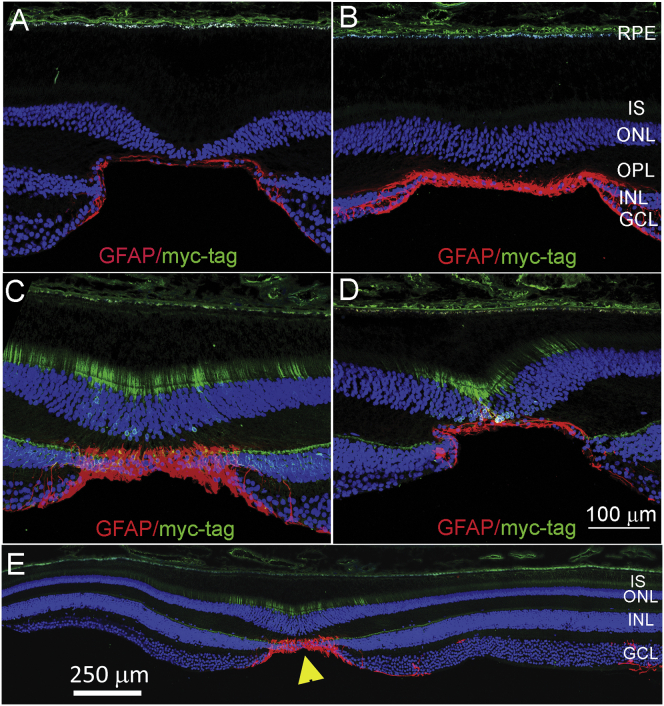
Immunohistochemistry Shows GFAP Labeling of Astrocytes in NHP Fovea (A–E) Immunofluorescence images of NHP fovea (A–D) and macula (E) with counterstaining using myc-tag (green) and glial fibrillary acidic protein (GFAP, red) antibodies, after dosing with excipient (A), or with AAV8-RS1/myc vector at 3e11 vg/eye (B, mid-vitreous injection; C and D, by para-retinal administration). (E) shows a larger area encompassing the fovea of eye in (C). GFAP is a marker for astrocytes and is detected at the surface of retina and limited principally to the center of the fovea (yellow arrowhead). The astrocyte region localizes to the region of AAV8-RS1/myc entry into the retina and expression in the foveal area. Scale bar (A–D) is 100 μm; (E) is 250 μm. Error bars are SEM.

Next, we explored whether para-retinal application would augment retinal expression in NHP eyes (Figure 2). Two NHP eyes were dosed by para-retinal application, a third was dosed by standard mid-vitreous injection, and a fourth received excipient as a control. Endogenous *RS1* normally expresses in the photoreceptor inner segments and at the post-synaptic dendritic input to bipolar cells (Figure 2, 1a–5a). Our AAV8-*RS1/myc* vector transgene contained a *myc* tag to distinguish viral RS1 expression from endogenous NHP *RS1*. Mid-vitreous injection showed no transgene expression (Figure 2, 1b), while both eyes receiving para-retinal application showed transgene *myc*-tagged *RS1* expression (Figure 2, 3b and 4b). These eyes dosed by para-retinal application also show expression at synaptic dendritic tips of bipolar cells and overlap with endogenous *RS1* (Figure 2, 5c); RS1 from vector expression also co-labels with *G*_*0*_α (Figure 2, 6c), a synaptic signaling protein localized in dendritic tips of ON bipolar cells.^11^^,^^12^ No transgene RS1 with *myc*-tag was identified in the retina with excipient control (Figure 2, 2b).

### Unique ILM in NHP Central Fovea

AAV8-RS1/myc vector expression was limited to the fovea of the two NHP eyes receiving para-retinal vector administration. This is not surprising, as it is known that normal AAV8 serotypes do not enter the retina freely after intravitreal application, although the barriers to vector penetration into the retina are poorly understood.^13^ We explored one of the unique features of foveal structure by looking at the glial component of the inner limiting membrane (ILM) for glial fibrillary acidic protein (GFAP, Figure 3, in red), a marker for astrocytes.^14^ All four eyes show GFAP localized to the fovea centralis (Figures 3A–3E) compared with the adjacent retinal area (Figure 3E), which corresponds to the region of transgene *hRS1* expression (green) in the two eyes dosed by para-retinal application (Figures 3C and 3D). This does not resolve why the AAV8-*RS1/myc* enters only the NHP central fovea, but it demonstrates that the expression pattern of the *myc*-tagged *RS1* coincides with the region of astrocyte GFAP expression in the fovea centralis and that this region is different than the surrounding macula.

## Discussion

Para-retinal application of vector is a practical method to increase local concentration of vector dosing of the retina without subjecting the tissue to surgical manipulation. In rabbit, the vector concentration remained greater near the retina even at 1 h after application. When tested in NHP eyes, para-retinal dosing gave greater transduction of the retina than did mid-vitreous injection. It is clinically routine to treat neovascular age-related macular degeneration by intravitreal injection of anti-VEGF antibodies (i.e., Avastin and Lucentis), which are nominally 150 kDa^15^. These antibodies are larger than many ocular drugs, but they are of considerably smaller size than an AAV virus (20–25 nm, 3,700 kDa), and the large AAV particle size impedes mixing through the vitreous to reach the retina at therapeutic levels. Additionally, AAV capsids have positively charged regions,^16^^,^^17^ which further impede mobility due to electrostatic interactions with the negatively charged vitreous along with hydrophobic interactions.^9^

The 6 mm vitreous space in rabbit presents experimental limitations for precise vector injections. We could position the needle tip reliably near the retina surface under direct observation by aiming for the optic disc; but injections are less reliable for the limited space in the anterior vitreous. Hence we designed the study to evaluate consequences of injecting vector near the retina surface and found that the vector does not freely mix and diffuse through the vitreous. The vector concentration remained greater near the retina even 1 h after para-retinal application. One expects that the limited AAV mobility observed in rabbit would be exacerbated further in the larger primate eye with even greater distance to the retina from the mid-vitreous injection site. In addition, the vitreous is a strongly hydrated extracellular matrix,^9^ which hinders vector mobility.^18^^,^^19^ Even relatively small molecules have limited diffusion through the human vitreous,^9^ and mobility decreases as the size of injected particles increases.

Preexisting neutralizing antibodies (NABs) compromise gene transfer.^20^ The high NAB serum titers are predictive of higher NAB levels in vitreous and correlate with diminished AAV vector expression in NHP eyes following intravitreal injection.^21^ Longer transit time of vector through the vitreous to reach the retina following intravitreal administration increases exposure time to vitreous NABs to neutralize the vector, and in turn this would reduce the effective vector dosing. Placing the vector adjacent to the retina surface provides for better kinetics and higher local vector concentration for retina uptake. As ocular inflammation after intravitreal AAV injection is dose dependent in X-linked retinoschisi (XLRS) human patients,^22^ a smaller overall injection dose that still maintains useful retinal delivery would reduce inflammation and other complications of ocular gene therapy.

This experiment demonstrated that para-retinal delivery increased the retinal expression compared with standard mid-vitreous delivery in NHP eyes. We confirmed that the retinal tropism of this AAV8-*hRS* in NHP fovea mirrors the expected distribution pattern of endogenous RS1. This is encouraging for considering the use of this technique to achieve greater transduction efficiency in human ocular clinical trials, which may be augmented further by using a novel vector capsid that has inherently better retinal transduction properties.

We demonstrated that the AAV8-RS1/myc vector gave limited extent of transduction of the NHP retina. First, we note that this is the retina of a normal NHP and not an XLRS disease animal. By comparison with mouse, AAV vectors enter the wild-type (WT) mouse retina poorly after intravitreal injection, whereas AAV8 vector enters the *Rs1*^−/y^ mouse retina (i.e., an “X-linked retinoschisis mouse” model) relatively easily and is expressed in photoreceptors.^23^ This indicates that *Rs1*^−/y^ pathology in some way alters the retina surface properties to augment vector entry, and this condition is not met by the normal NHP retina. Accordingly, we would not necessarily expect the limitations of a WT NHP to correspond to transgene expression in XLRS human disease, which is our current disease target for human ocular gene therapy. Further, translating these results to human application would be subject to the considerable heterogeneity of human vitreous due to liquefaction from age and retinal pathology.^24^^,^^25^ We expect to employ para-retinal vector application for future participants in our clinical gene therapy trial for XLRS disease (ClinicalTrials.gov #NCT02317887).

## Materials and Methods

### Animals

Male New Zealand white rabbits (6–8 months of age), and male Cynomolgus NHPs (5–6 years of age) were used for the study. Research was conducted in accord with the ARVO Statement on the Use of Animals in Ophthalmic and Vision Research, and it was approved by the Animal Care and Use Committee of the National Eye Institute, NIH. Rabbits were purchased from Covance (Covance, Princeton, NJ, USA) and reared under normal cyclic 12:12 h lighting. The NHP studies were conducted at Charles River Laboratories (Mattawan, MI, USA) under protocols approved by the Charles River Institutional Animal Care and Use Committee.

### AAV8-hRSP-*hRS/myc* Vector Construct

A DNA fragment of normal *hRS1* cDNA plus the *myc* tag sequence was synthesized and inserted into pUC57 plasmid and named pUC57-*RS1/2myc*. Fragments were obtained by SphI and Xho I restriction enzyme digests and inserted into Sph I and Xho I sites of the pscAAV8-RSP/IRBP-*hRS*.^10^ This construct was packaged in AAV8 by the NEI core facility as described previously.^10^

### Vector Partitioning in Rabbit Vitreous 1 h after Para-retinal Injection

The AAV8-CMV-*EGFP* vector (1.5e11 vg/eye) was administered to 15 eyes of New Zealand white rabbits by deep intravitreal injection with direct observation as the needle tip was positioned close to the retinal surface, termed para-retinal injection. Rabbits were first sedated with ketamine (35 mg/kg) and xylazine (5 mg/kg), and pupils were dilated fully with 10% phenylephrine and 1% tropicamide. The injection site was prepared with 5% povidone iodine. Injections were performed using a 1/2-inch long 28 gauge (G) needle (Becton Dickinson Company, Franklin Lakes, NJ, USA) inserted 3 mm posterior to the limbus in the supero-temporal quadrant under direct visualization with an operating microscope (Zeiss Lumera microscope, Jena, Germany) equipped with the Alcon NGENUITY Visualization System (Alcon, Fort Worth, TX, USA). The needle tip was advanced close to the retina surface, positioned bevel down over the optic disc, and the vector solution was injected slowly while observing vector pooling at the posterior pole. This was performed on 3 sets of rabbits on separate days, and all rabbits that were injected by the para-retinal method were included in the analysis.

### AAV8 Vector Titer in Anterior and Posterior Rabbit Vitreous

1 h after injections, rabbits were induced with general anesthesia, then euthanized with Beuthanasia-D by intravascular (IV) at 85 mg/kg or by intracardiac (IC) at 1 mL/10 lbs. The eye globe was separated from surrounding tissues and was placed into a 50 mL conical tube, which was immersed in liquid nitrogen for 20 min. The eye was transferred to a box filled with dry ice. Subsequently, the frozen vitreous was separated intact from the ocular tissues as follows: the eye globe was placed on a cold metal plate, incisions were made onto the posterior sclera with a cold scalpel, and the choroid and retina were separated from the frozen vitreous with forceps. The frozen vitreous was hemisected with the cold scalpel, and anterior and posterior vitreous samples were placed in separate 15 mL tubes. Quantitative PCR (qPCR) was performed to determine the AAV8 vector titer for each hemisected vitreous segment: 10 μL aliquots of each segment were treated with DNase I in a 50 μL reaction volume and inactivated the DNase I enzyme by adding 50 μL of 200 mM EDTA. 10 μL reaction mixture was transferred into 990 μL of qPCR dilution buffer, and 5 μL of the diluted sample was used for qPCR in a total reaction volume of 25 μL. The plasmid DNA of CMV-*EGFP* digested with SmaI was used as a standard curve to calculate the copy number of each vitreous sample.

### Statistical Analysis

Two-tail Wilcoxon matched pairs signed rank test was used to do the statistical analysis (GraphPad prism, San Diego, CA, USA). p values smaller than 0.05 on two-tail test were considered a significant difference.

### Para-Retinal Injection with NHP

NHPs were screened prior to injection for neutralizing antibodies against AAV8 serotype, and NHPs having AAV8 NAB titer <10 were used for this study. NHPs were maintained under general anesthesia with isoflurane gas and positioned in dorsal recumbency. Topical ocular proparacaine (proparacaine hydrochloride 0.5%) was applied, and the eyelid margins and conjunctiva were cleaned with 5% betadine solution. An eyelid speculum was placed, and a lateral canthotomy was performed. For the first NHP, 100 μL aqueous paracentesis was performed with a 31G needle to control intraocular pressure, and then 3e11 vg in 100 μL of AAV8 vector was injected into the right eye and 100 μL excipient (10 mM Tris-HCl, 180 mM NaCl, 0.001% pluronic F68) was injected into the left eye using an 8 mm 31 g BD insulin syringe/needle inserted 3 mm posterior to the limbus. The needle tip was advanced to the mid-vitreous with the tip rotated toward the retina. Both eyes of the second NHP received 3e11 vg in 100 μL AAV8 vector administered near the retina by para-retinal injection. The globe was stabilized with conjunctival forceps, and two 25G valved scleral ports were placed 3 mm posterior to the limbus. Paracentesis was performed with a 31G needle to remove 100 μL aqueous to control intraocular pressure. A flat corneal vitrectomy lens was placed to visualize the fundus. A 25G illumination probe was inserted through the superior port, and a 25G/38G single bore injection cannula (PolyTip #3219; MedOne, Sarasota, FL, USA) was inserted through the inferior port. The cannula tip was positioned over the fovea immediately adjacent to the retina, and 100 μL AAV8 vector was delivered slowly to the para-retinal location. The injections were performed using a foot-pedal control to inject slowly over approximately 1 min. Care was taken not to penetrate the retina, and no subretinal bleb was created. Animals were maintained in dorsal recumbency for 15 min after vector application.

All NHP animals received 40 mg methylprednisolone acetate intramuscular (IM) the day prior to vector dosing. The NHP receiving mid-vitreous vector application required a booster injection of 40 mg methylprednisolone acetate on day 8 due to mild uveitis and then received weekly IM doses for weeks 2–8 when the uveitis was completely controlled. The para-retinal injected animal received weekly 40 mg methylprednisolone acetate IM doses for weeks 4–6 to alleviate mild uveitis until the problem was completely controlled.

### Preparation of NHP Retina Sections

The NHP were euthanized via overdose of sodium pentobarbital 10 weeks post injection. Eyes were marked for orientation; a slit was made at the pars plana on the temporal side, and 0.5 mL 4% paraformaldehyde solution (PFA) was injected into the vitreous 2 mm posterior to the nasal limbus. Eyes were enucleated and placed in iced 4% PFA for 4 h, and the cornea, lens, and vitreous were removed to leave the posterior eyecup. The eyecup was continuously fixed overnight in 2% PFA and then cryoprotected in gradient sucrose concentrations to a final 30% in 3 days. The eyecup was embedded in optimal cutting temperature compound, and horizontal retinal serial cryosections (12 μm) were cut using a research cryostat (Leica Biosystems, Buffalo Grove, IL, USA) beginning at the inferior margin of the eye. After every 6 to 8 sections, two sections were collected and saved for evaluating transgene expression.

### Immunofluorescent Assay

The immunofluorescent assay was performed as described by Zeng et al.^23^ Briefly, retinal sections were rinsed in 0.1% Triton X-100 in PBS and preincubated with PBS containing 20% normal goat serum (Sigma, Steinheim, Germany) and 0.5% Triton X-100 at room temperature (RT) for 2 h to permeabilize the tissue and block nonspecific antibody binding. *Myc*-tag counterstaining was used to distinguish the *myc*-tagged transgene hRS1 protein from endogenous RS1. Cell markers were incubated overnight at 4°C with primary antibodies (Table 1) diluted in washing buffer. Sections were washed and incubated at RT for 1 h with appropriate secondary antibodies conjugated to red-fluorescent Alexa Fluor 568 dye or green-fluorescent Alexa Fluor 488 dye (Invitrogen, Carlsbad, CA, USA). Nuclei were stained with DAPI, and sections were mounted with Fluorogel. Retinal images were captured and processed using a Nikon C2 confocal microscope with Advance Element software (Nikon, Tokyo, Japan). Image analysis was performed by using Photoshop CS6 image-editing software (Adobe Systems, San Jose, CA, USA).

**Table 1:** List of Primary Antibodies Used in Immunofluorescent Assay

Name	Host	Dilution	Product Source
RS1	guinea pig	1:1,000	customized antibody, Thermo Fisher Scientific, Waltham, MA, USA
Myc-tag	rabbit	1:1,000	2272S, Cell Signaling Technology, Danvers, MA, USA
G_0_α	mouse	1:500	MAB3070, Millipore Corporation, Bedford, MA, USA
GFAP	mouse	1:1,000	G3893, Sigma-Aldrich, St. Louis, MO, USA

## Author Contributions

Y.Z. designed and conducted the experiments and wrote the paper; R.B. conducted the experiments; J.B. conducted the experiments; H.E.W. conducted the experiments and paper review; D.M. conducted the experiments; L.L.W. coordinated the research activity planning and execution and paper review; P.A.S. designed the experiments and wrote the paper.

## Conflicts of Interest

The authors declare no competing interests.
